# A Novel Diagnostic Nomogram for Noninvasive Evaluating Liver Fibrosis in Patients with Chronic Hepatitis B Virus Infection

**DOI:** 10.1155/2020/5218930

**Published:** 2020-06-02

**Authors:** Danying Cheng, Gang Wan, Lei Sun, Xiaomei Wang, Weini Ou, Huichun Xing

**Affiliations:** ^1^Center of Liver Disease, Beijing Ditan Hospital Capital Medical University, Beijing 100015, China; ^2^Statistics Room, Beijing Ditan Hospital Capital Medical University, Beijing 100015, China; ^3^Department of Pathology, Beijing Ditan Hospital Capital Medical University, Beijing 100015, China

## Abstract

**Objective:**

To establish a novel nomogram for diagnosing liver fibrosis in patients with chronic hepatitis B virus (HBV) infection and verify the diagnostic performance of the established nomogram.

**Methods:**

Patients with chronic HBV infection who met the inclusion and exclusion criteria were enrolled in this retrospective study; 70% and 30% of patients were randomly assigned to training dataset and validation dataset, respectively. The risk factors for liver fibrosis were screened using the univariate and multivariate logistic regression analyses. Based on the results, a nomogram was established and verified.

**Results:**

508 patients with chronic HBV infection were included in this study (*n* = 355 for training dataset and *n* = 153 for validation dataset). The logistic regression analysis showed that liver stiffness measurement (LSM), platelet (PLT) count, and prothrombin time (PT) were independent risk factors for liver fibrosis (*P* < 0.01), which were used to establish the nomogram. The consistency index (C-index) of the nomogram established for diagnosing liver fibrosis was 0.875. The calibration line and the ideal line were consistent, which indicated that diagnosis of liver fibrosis by the established model was accurate. The values of area under the receiver operator characteristic (ROC) curve (AUROC) for diagnosing liver fibrosis by the nomogram were 0.857 and 0.862 in the training dataset and validation dataset, respectively, which were noticeably higher than those in the well-known serological models, including the aspartate aminotransferase- (AST-) to-platelet ratio index (APRI) scoring model, fibrosis-4 (FIB-4) scoring model, APAG model (including age, PT, albumin, and *γ*-glutamyl transferase), and S-index model (all *P* < 0.05).

**Conclusion:**

LSM, PT, and PLT were found as independent risk factors for liver fibrosis. The established nomogram exhibited an excellent diagnostic performance, and it can more visually and individually evaluate the probability of liver fibrosis in patients with chronic HBV infection.

## 1. Introduction

Liver fibrosis is an inevitable stage of development of chronic hepatitis B (CHB) to hepatic cirrhosis. Fibrosis assessment during antiviral treatment is a key step in antiviral therapy evaluation. These noninvasive approaches for assessment of liver fibrosis include various biochemical serum markers, or imaging modalities that provide a physical measure of liver stiffness [1]. To date, a number of serological diagnostic models have been presented, such as the aspartate aminotransferase- (AST-) to-alanine aminotransferase (ALT) ratio (AAR), AST-to-platelet ratio index (APRI), and fibrosis-4 (FIB-4). However, there is no unified standard for developing serological diagnostic models; thus, the reliability of diagnosis has still remained a main challenge. In addition, due to the difference in spectrum of disease, several diagnostic models were established and verified for patients with chronic hepatitis C virus infection. However, further studies need to be conducted to indicate whether those diagnostic models are applicable to patients with chronic hepatitis B virus (HBV) infection. Patients with chronic HBV infection with complete clinical and pathological data were enrolled in this retrospective study to screen the risk factors for liver fibrosis, which were used to establish a diagnostic nomogram applicable to chronic HBV patients in China.

## 2. Study Subjects and Methods

### 2.1. Study Subjects

This was a single-center, retrospective study. Patients who were infected with chronic HBV and received liver biopsy at Beijing Ditan Hospital, Capital Medical University (Beijing, China), during January 2015 and July 2016 were recruited into this research. Inclusion criteria were as follows: (1) patients who met the diagnostic standard presented in 2017 Clinical Practice Guidelines on the management of HBV infection by the European Association for the Study of the Liver (EASL) [2]; (2) those with history of CHB-positive or hepatitis B surface antigen- (HBsAg-) positive CHB for over 6 months; (3) patients aged 18-65 years old; (4) no gender limitation; (5) patients with ALT level less than 10 times of upper limit of normal (ULN) range; and (6) those with total bilirubin (TBIL) level less than 5 times of ULN. Exclusion criteria were as follows: (1) patients with several types of hepatitis viruses, including types A, C, D, and E; (2) patients who were infected with human immunodeficiency virus (HIV); (3) hepatic histology conformed to other pathogeneses of chronic liver disease (except for fatty liver); (4) patients with non-HBV viruses, such as autoimmune hepatitis (AIH), primary biliary cholangitis (PBC), primary sclerosing cholangitis (PSC), inherited metabolic liver disease, drug-induced liver injury (DILI), and alcoholic liver disease; (5) patients with hepatocellular carcinoma (HCC); and (6) those with incomplete clinical data.

### 2.2. Data Collection

Patients' clinical data were collected, including general demographic characteristics, medical history, as well as results of blood routine examination, coagulation function test, liver function test, and liver stiffness measurement (LSM). The tests were carried out one week before and after undergoing fine-needle aspiration biopsy of liver. The LSM was performed by FibroScan (Echosens, France) with the M probe in all patients. During the measurement, the patient was placed in supine position with the right hand behind the head, exposing the right intercostal space, making the large right lobe of the liver accessible. The area surrounded by the horizontal line of xiphoid process, the right midaxillary line, and the lower margin of the ribs were taken as the detection area into account. The probe was vertically pressed against the skin, the measurement position was selected in the intercostal space, and the probe button was pressed to collect the image and obtain the measured value. Herein, 10 valid measurements (VMs) were obtained repeatedly (the success rate of operation was ≥70%), and the median number was taken as the results. Besides, 70% and 30% of patients were randomly allocated to training dataset and validation dataset, respectively.

### 2.3. Evaluation of Liver Fibrosis

Liver biopsy specimens with 19 mm in length and 1.6 mm in diameter were obtained using 16G disposable needles (Bard Monopty Disposable Biopsy System; Bard, Inc., Murray Hill, NJ, USA). Among 98.8% of patients, there were at least 11 portal tracts for evaluating liver fibrosis. Liver fibrosis was staged according to the METAVIR scoring system [3]: F0 (no fibrosis), F1 (portal fibrosis without septa), F2 (portal fibrosis with limited septa), F3 (numerous septa without cirrhosis), and F4 (cirrhosis). The degree of fibrosis F0-F1 was defined as no significant liver fibrosis, F2-F4 as significant liver fibrosis [2].

### 2.4. Statistical Analysis

The SPSS 19.0 software (IBM, Armonk, NY, USA) and R 3.02 programming language were used to carry out statistical analyses. The continuous variables were expressed as mean ± standard deviation (SD) or median (interquartile range (IQR)). The differences between groups were compared by *t*-test or the nonparametric rank-sum test. The categorical variables were described by frequency, and differences between groups were compared by *χ*^2^ test or Fisher's exact test. Univariate and multivariate logistic regression analyses were undertaken to quantitatively analyze the effects of each factor on dependent variable in training dataset. The screened independent risk factors were imported into the R software to establish the diagnostic nomogram and calculate the consistency index (C-index). The Bootstrap method was adopted to carry out internal verification for validation dataset. Sampling was repeated for 1000 times to draw a calibration graph to compare the diagnostic nomogram with grades of liver fibrosis graded by the METAVIR scoring system. The diagnostic values of the established nomogram and four well-known noninvasive diagnostic models were compared via area under the receiver operating characteristic (ROC) curve (AUROC). *P* < 0.05 was considered statistically significant.

## 3. Results

### 3.1. Comparing Patients' Baseline Demographic Characteristics and Clinical and Pathological Data between Training Dataset and Validation Dataset

A total of 508 patients with chronic HBV infection were enrolled in the current study, including 334 male patients and 174 female patients (range of age: 18-63 years old; median age, 39.71 ± 9.97 years old). In addition, 355 and 153 patients were assigned to training dataset and validation dataset, respectively. The results of statistical analysis showed that the differences in gender, age, and clinical and pathological data between the two datasets were not statistically significant (*P* > 0.05) (Table 1).

### 3.2. Logistic Regression Analysis of Liver Fibrosis in Training Dataset

The 355 patients in training dataset were assigned to the nonsignificant liver fibrosis group (209 cases) and significant liver fibrosis group (146 cases) according to grade of liver fibrosis graded by the METAVIR scoring system. The results unveiled that there were significant differences in age, LSM value, AST level, directed bilirubin (DBIL) level, alkaline phosphatase (ALP) level, *γ*-glutamyl transferase (GGT), cholinesterase (CHE), albumin (ALB) level, platelet (PLT) count, and prothrombin time (PT) between the two groups (*P* < 0.05) (Table 2). In the multivariate logistic regression analysis, backward linear regression was adopted to screen the variables; when *P* > 0.05, the variables were eliminated. Three variables, including LSM, PLT, and PT, were eventually found as independent factors influencing liver fibrosis (Table 3).

### 3.3. Establishment and Verification of the Diagnostic Nomogram and Evaluation of Its Diagnostic Performance

**Figure 1:**
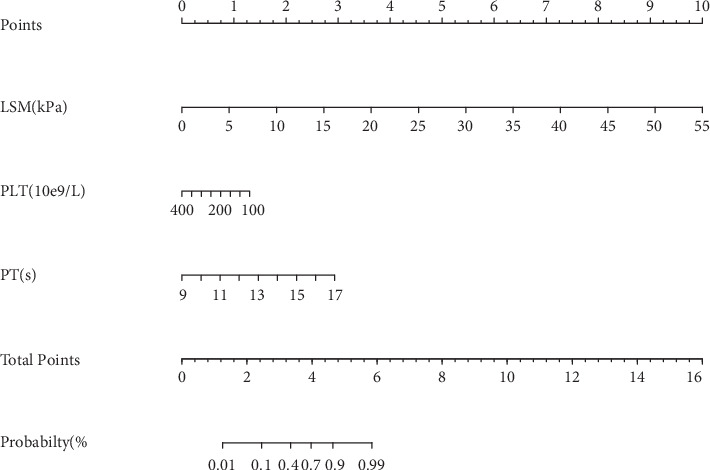
The nomogram established for diagnosing liver fibrosis. To use the nomogram, the value of an individual patient is located on each variable axis, and a line is drawn upward to determine the number of points received for the value of each variable. The sum of these numbers is located on the total point axis, and a line is drawn downward to the probability axis to determine the likelihood of diagnosing liver fibrosis.

With consideration of significant liver fibrosis as a dependent variable, and LSM, PLT, and PT as independent variables, the R programming language was adopted to establish the nomogram for diagnosis of liver fibrosis (Table 4, Figure 1). Each variable received a score. With summation of the total score of all the variables and locating it to the total point scale, we could determine the probabilities of the outcomes by drawing a vertical line to the total score. The range of total score was 1.0-6.0, and the range of corresponding risk rate was 0.01-0.99. A higher total score indicated a higher probability of diagnosis of liver fibrosis. The C-index of the nomogram for diagnosing liver fibrosis was 0.875 (95% confidence interval (CI): 0.818-0.932), highlighting high accuracy of the nomogram.

**Figure 2:**
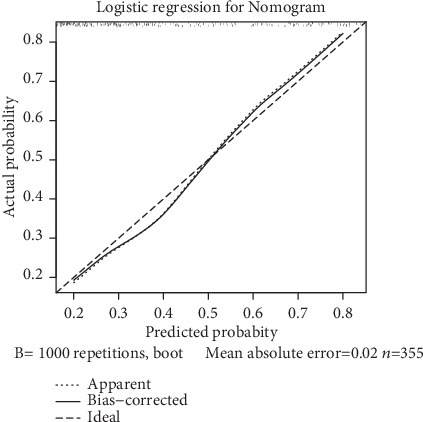
Calibration graph for comparing the established nomogram with grades of liver fibrosis graded by the METAVIR scoring system.

The Bootstrap method was adopted for internal verification in validation dataset; sampling was repeated for 1000 times, and the average error probability was 0.022; in the calibration graph, the calibration line was well fitted with the ideal line, indicating that the distinction degree of the nomogram established for diagnosis of liver fibrosis was remarkable (Figure 2).

### 3.4. Comparing Diagnostic Values of the Nomogram with Four Well-Known Serological Models

**Figure 3:**
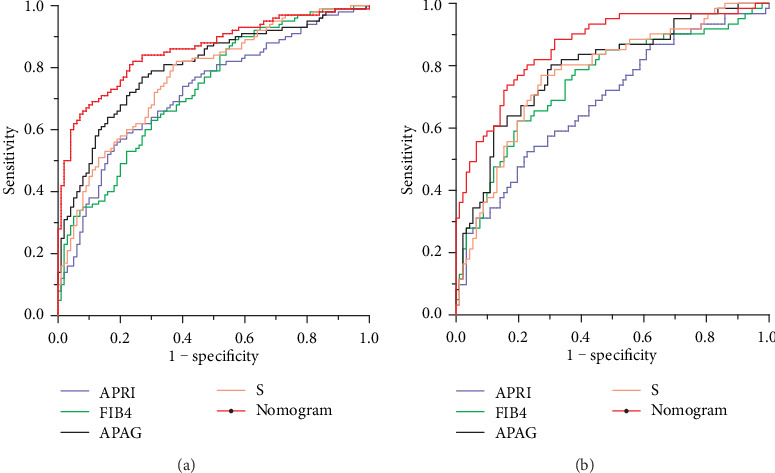
ROC curves of the established nomogram and four well-known serological models for diagnosing liver fibrosis. (a) Training dataset (*n* = 355). (b) Validation dataset (*n* = 153).

The data of patients in training dataset and validation dataset were imported into the nomogram, APRI scoring model, FIB-4 scoring model, APAG model (including age, PT, ALB, and GGT), and S-index model to draw the ROC curve and compare diagnostic values of the nomogram and four well-known serological models. The results unveiled that the AUROC values for diagnosing liver fibrosis by the nomogram in training dataset and validation dataset were 0.857 (95% CI: 0.816-0.899) and 0.862 (95% CI: 0.801-0.922), respectively, which were significantly higher than those in the APRI scoring model, FIB-4 scoring model, APAG model, and S-index model (all *P* < 0.05). This indicated that the diagnostic performance of the established nomogram was markedly higher than that of the four above-mentioned serological models (Table 5, Figure 3).

## 4. Discussion

HBV infection seriously threatens public health worldwide. China is one of the area with high incidence of CHB. Liver fibrosis is an inevitable stage of development of CHB to hepatic cirrhosis. Accurate and timely diagnosis of liver fibrosis are of great importance for effective therapy. At present, liver biopsy is still the “gold standard” [8] for diagnosing liver fibrosis. However, liver biopsy has not been popularized among patients, restricting its clinical application. In recent years, noninvasive diagnostic methods have been increasingly applied for liver fibrosis [9–12]. Transient elastography (TE) is an imaging technique employing ultrasound monitoring of the passage of a low-frequency pressure wave through liver tissue and has been shown effective for the detection of liver fibrosis [13, 14]. Additionally, a number of scholars suggested that the combination of TE and serological indexes can promote diagnostic performance [15, 16]. However, a limited number of studies have concentrated on the establishment of noninvasive diagnostic models for liver fibrosis by the combination of diagnostic imaging and serological indexes.

A total of 508 patients with chronic HBV infection were enrolled in the present research, and 70% and 30% of them were randomly assigned to training dataset and validation dataset, respectively. Univariate and multivariate logistic regression analyses were carried out on clinical and pathological data of training dataset. The results unveiled that three variables (LSM, PT, and PLT) were independent risk factors for liver fibrosis. Previous researches demonstrated that TE has a high diagnostic value for patients with liver fibrosis [2, 17]. PT is an important indicator reflecting the comprehensive activity of exogenous coagulation factors, and the main coagulation factors involved in coagulation process, such as factors II, V, VII, and X. In case of liver inflammation and hepatic necrosis, due to the reduction of synthesis of the coagulation factors, PT will be prolonged. The half-life of the coagulation factors is very short (i.e., half-life of factor VII is only 4-6 h). Therefore, PT is often used as a sensitive indicator of the severity of liver injury, and it is one of the important indicators for diagnosing liver failure. In fact, PT can be prolonged due to insufficient synthesis of coagulation factors. Fibrosis can be used to describe the pathological state of excess deposition of fibrous tissue, as well as the process of connective tissue deposition in healing. In the process of liver fibrosis, the number of effective hepatocytes may be reduced, which may decrease the synthesis of coagulation factors and also result in the prolongation of PT. In the present study, the liver inflammation and hepatic necrosis were mild (the mean levels of ALT, AST, and TBIL were 49.23 IU/L, 34.54 IU/L, and 14.18 *μ*mol/L, respectively). Therefore, it could be speculated that the change of PT was mainly caused by liver fibrosis. This explained that why PT was one of the independent factors influencing liver fibrosis in the multivariate regression analysis. However, it is noteworthy that liver inflammation and hepatic necrosis may influence the outputs of the model evaluating liver fibrosis.

Although patients with cirrhosis may have platelet dysfunction, it is not typically associated with serious bleeding. Based on LSM, PT, and PLT, a nomogram for diagnosing liver fibrosis was established in the current study. The C-index represented the diagnostic performance of the nomogram, and a calibration graph was used to visually observe its performance in diagnosing liver fibrosis. Our findings revealed that the C-index of the established nomogram was 0.875. The Bootstrap method was adopted for internal verification. The calibration line was close to the ideal line, which indicated high accuracy of the nomogram. The AUROC values of the established nomogram were 0.857 and 0.862 in training dataset and validation dataset, respectively, which were noticeably higher than those in the APRI scoring model, FIB-4 scoring model, APAG model, and S-index model (all *P* < 0.05). This demonstrated that the diagnostic performance of the nomogram was markedly higher than that of the four serological models.

Nomogram was initially presented by Kattan et al. in 1998 and has been used for predicting prostate cancer recurrence after radical prostatectomy [18]. Nomograms from multivariable logistic models are used as a graphical user interface to display the predicted probabilities of an event [19]. Nomograms are frequently used to estimate prognosis in oncology [20–32]. Gao et al. developed a prognostic nomogram to assess the individual prognosis for CHB patients. Their developed nomogram outperformed other traditional models [33, 34]. A number of scholars have applied nomograms to predict risk factors for sleep apnea, diabetes, acute myocardial infarction, etc. [35–38]; however, application of a nomogram for noninvasive diagnosis of liver fibrosis has been scarcely reported. The nomogram established in the present study for noninvasive diagnosis of liver fibrosis exhibited a remarkably superior diagnostic performance compared with traditional models. It could realize individualized diagnosis by calculating the probability of liver fibrosis for every patient, which may assist physicians to present more effective therapies for liver fibrosis.

## 5. Conclusions

The results of the present study uncovered that LSM, PT, and PLT were independent risk factors for liver fibrosis. The established nomogram combined LSM values with serological indexes, leading to present an accurate and individualized diagnostic method with high accuracy for liver fibrosis, which is worthy of application in clinical trials. However, in spite of novelties of the present study, further studies with large sample size need to be conducted to confirm our findings and to develop more effective noninvasive diagnostic methods for liver fibrosis using nomograms.

## Figures and Tables

**Table 1:** Comparing patients' baseline demographic characteristics and clinical and pathological data between training dataset and validation dataset.

Characteristics	Training dataset	Validation dataset	*P*
	*n* = 355 (%)	*n* = 153 (%)	
Gender			
Male	237 (66.76)	97 (63.40)	0.464
Female	118 (33.24)	56 (36.60)	
Age (yrs)	39.77 ± 10.06	39.56 ± 9.81	0.828
Significant fibrosis			
No	209 (58.87)	92 (60.13)	0.791
Yes	146 (41.13)	61 (39.87)	
LSM (kPa)	9.24 ± 6.91	8.66 ± 5.03	0.293
Laboratory data			
ALT (IU/L)	48.77 ± 44.69	50.31 ± 49.54	0.730
AST (IU/L)	34.12 ± 23.08	35.50 ± 25.39	0.549
TBIL (*μ*mol/L)	14.30 ± 6.70	13.90 ± 6.87	0.541
DBIL (*μ*mol/L)	4.95 ± 3.85	4.76 ± 2.52	0.520
ALP (IU/L)	71.49 ± 21.61	72.08 ± 20.93	0.773
GGT (IU/L)	34.01 ± 40.09	35.05 ± 42.73	0.793
CHE (IU/L)	8523.92 ± 2110.18	8662.37 ± 2426.23	0.540
TP (g/L)	75.87 ± 5.75	75.83 ± 6.61	0.951
ALB (g/L)	46.03 ± 4.00	46.47 ± 4.45	0.277
GLO (g/L)	29.68 ± 5.01	29.51 ± 4.46	0.721
WBC (×10^9^/L)	5.69 ± 1.53	5.78 ± 1.56	0.541
RBC (×10^12^/L)	4.91 ± 0.52	4.82 ± 0.50	0.067
HGB (g/L)	150.45 ± 18.06	148.33 ± 17.79	0.224
PLT (×10^9^/L)	178.71 ± 57.84	189.46 ± 57.34	0.055
PT (S)	11.87 ± 0.91	11.86 ± 0.88	0.892

Data were presented as *n* (%), mean ± standard deviation, or median (interquartile range). LSM: liver stiffness measurement; ALT: alanine aminotransferase; AST: aspartate aminotransferase; TBIL: total bilirubin; DBIL: directed bilirubin; ALP: alkaline phosphatase; GGT: *γ*-glutamyl transferase; CHE: cholinesterase; TP: total protein; ALB: albumin; GLO: globulin; WBC: white blood cell; RBC: red blood cell; HGB: hemoglobin; PLT: platelet count; PT: prothrombin time.

**Table 2:** Comparison of patients' baseline characteristics and clinical data between significant liver fibrosis and nonsignificant liver fibrosis groups in training dataset.

Characteristics	Nonsignificant liver fibrosis	Significant liver fibrosis	*P*
	*n* = 209 (%)	*n* = 146 (%)	
Gender			
Male	134 (64.11)	103 (70.55)	0.205
Female	75 (35.89)	43 (29.45)	
Age (yrs)	38.80 ± 10.54	41.16 ± 9.18	0.030
LSM (kPa)	6.64 ± 2.35	12.95 ± 9.22	<0.001
Laboratory data			
ALT (IU/L)	46.95 ± 40.90	51.37 ± 49.64	0.376
AST (IU/L)	31.27 ± 19.14	38.20 ± 27.33	0.009
TBIL (*μ*mol/L)	13.70 ± 5.43	15.16 ± 8.13	0.059
DBIL (*μ*mol/L)	4.35 ± 1.92	5.80 ± 5.44	0.002
ALP (IU/L)	68.27 ± 18.02	76.09 ± 25.27	0.001
GGT (IU/L)	25.29 ± 22.67	46.49 ± 54.04	<0.001
CHE (IU/L)	8844.54 ± 2150.82	8064.95 ± 1968.48	0.001
TP (g/L)	76.07 ± 5.65	75.58 ± 5.90	0.438
ALB (g/L)	46.54 ± 3.87	45.30 ± 4.08	0.004
GLO (g/L)	29.26 ± 5.04	30.28 ± 4.91	0.059
WBC (×10^9^/L)	5.76 ± 1.52	5.58 ± 1.54	0.260
RBC (×10^12^/L)	4.91 ± 0.52	4.91 ± 0.52	0.901
HGB (g/L)	150.75 ± 18.27	150.01 ± 17.80	0.703
PLT (×10^9^/L)	196.94 ± 49.34	152.62 ± 59.25	<0.001
PT (S)	11.57 ± 0.67	12.29 ± 1.04	<0.001

**Table 3:** Univariate and multivariate logistic regression analyses of liver fibrosis in training dataset.

	Univariate analysis		Multivariate analysis	
	OR (95% CI)	*P*	OR (95% CI)	*P*
Gender	0.746(0.473~1.175)	0.206		
Age (yrs)	1.024 (1.002~1.046)	0.031		
LSM (kPa)	1.533 (1.373~1.712)	<0.001	1.439 (1.288~1.608)	<0.001
Laboratory data				
ALT (IU/L)	1.002 (0.997~1.007)	0.362		
AST (IU/L)	1.015 (1.003~1.026)	0.010		
TBIL (*μ*mol/L)	1.034 (1.000~1.069)	0.050		
DBIL (*μ*mol/L)	1.205 (1.091~1.33)	<0.001		
ALP (IU/L)	1.018 (1.007~1.029)	0.001		
GGT (IU/L)	1.023 (1.012~1.034)	<0.001		
CHE (IU/L)	1.000 (1.000~1.000)	0.001		
TP (g/L)	0.985 (0.95~1.023)	0.437		
ALB (g/L)	0.923 (0.874~0.976)	0.005		
GLO (g/L)	1.043 (0.998~1.09)	0.061		
WBC (×10^9^/L)	0.922 (0.802~1.061)	0.260		
RBC (×10^12^/L)	0.974 (0.648~1.465)	0.900		
HGB (g/L)	0.998 (0.986~1.009)	0.702		
PLT (×10^9^/L)	0.984 (0.98~0.989)	<0.001	0.993 (0.987~0.998)	0.006
PT (S)	3.036 (2.191~4.207)	<0.001	2.085 (1.409~3.085)	<0.001

**Table 4:** Point assignment from nomograms and prognostic scores.

LSM (kPa)	Points	PLT (×10^9^/L)	Points	PT (S)	Points	Total points	*P*
0	0.0	50	1.4	9	0.0	1.0	0.01
5	0.9	100	1.2	10	0.4	2.0	0.10
10	1.8	150	1.0	11	0.7	3.0	0.30
15	2.7	200	0.8	12	1.1	4.0	0.50
20	3.6	250	0.6	13	1.5	4.5	0.70
25	4.6	300	0.4	14	1.8	5.0	0.95
30	5.5	350	0.2	15	2.2	6.0	0.99
35	6.4	400	0.0	16	2.6		
40	7.3			17	2.9		
45	8.2						
50	9.1						
55	10.0						

**Table 5:** Comparing AUROC values of different models.

	AUROC	SE	*P*	95% CI for AUROC		*χ* ^2^	*P*
				Lower	Upper		
Training dataset							
APRI scoring model	0.722	0.028	<0.001	0.668	0.777	31.02	<0.001
FIB-4 scoring model	0.729	0.027	<0.001	0.677	0.781	24.63	<0.001
APAG model	0.800	0.025	<0.001	0.752	0.848	7.79	0.005
S-index model	0.770	0.025	<0.001	0.721	0.819	13.21	<0.001
The nomogram	0.857	0.021	<0.001	0.816	0.899	—	—
Validation dataset							
APRI scoring model	0.687	0.044	<0.001	0.600	0.773	19.96	<0.001
FIB-4 scoring model	0.749	0.042	<0.001	0.668	0.831	7.56	0.006
APAG model	0.791	0.038	<0.001	0.717	0.865	4.53	0.033
S-index model	0.772	0.039	<0.001	0.695	0.848	5.61	0.018
The nomogram	0.862	0.031	<0.001	0.801	0.922		

The four serological diagnostic models are formulated as follows [4–7]: APRI = [AST(IU/L)/ULN]/PLT(10^9^/L) × 100, FIB − 4 = [age (years) × AST(IU/L)]/[PLT(10^9^/L) × ALT(g/L)^1/2^], APAG = *e*^*p*^/(1 + *e*^*p*^), *P* = −9.340 + 0.997 × ln[age(years)] + 6.355 × ln[PT(s)] − 3.372 × ln[ALB(g/L)] + 0.677 × ln[GGT(IU/L)], and S − index = [1000 × GGT(IU/L)]/[PLT(10^9^/L) × ALB(g/L)^2^].

## Data Availability

The processed data used to support the findings of this study are included within the article. The original data can be available from the corresponding author upon request.

## References

[1] Clark P. J., Patel K. (2011). Noninvasive tools to assess liver disease. *Current Opinion in Gastroenterology*.

[2] Lampertico P., Agarwal K., Berg T. (2017). EASL 2017 Clinical Practice Guidelines on the management of hepatitis B virus infection. *Journal of Hepatology*.

[3] Bedossa P., Poynard T. (1996). An algorithm for the grading of activity in chronic hepatitis C. *Hepatology*.

[4] Wai C. T., Greenson J. K., Fontana R. J. (2003). A simple noninvasive index can predict both significant fibrosis and cirrhosis in patients with chronic hepatitis C. *Hepatology*.

[5] Sterling R. K., Lissen E., Clumeck N. (2006). Development of a simple noninvasive index to predict significant fibrosis in patients with HIV/HCV coinfection. *Hepatology*.

[6] Cheng J., Hou J., Ding H. (2015). Validation of ten noninvasive diagnostic models for prediction of liver fibrosis in patients with chronic hepatitis B. *PLoS One*.

[7] Zhou K., Gao C.-F., Zhao Y.-P. (2010). Simpler score of routine laboratory tests predicts liver fibrosis in patients with chronic hepatitis B. *Journal of Gastroenterology and Hepatology*.

[8] Germani G., Hytiroglou P., Fotiadu A., Burroughs A. K., Dhillon A. P. (2011). Assessment of fibrosis and cirrhosis in liver biopsies: an update. *Seminars in Liver Disease*.

[9] Bedossa P. (2009). Assessment of hepatitis C: non-invasive fibrosis markers and/or liver biopsy. *Liver International*.

[10] Hagan M., Asrani S. K., Talwalkar J. (2015). Non-invasive assessment of liver fibrosis and prognosis. *Expert Review of Gastroenterology & Hepatology*.

[11] Van Beers B. E., Daire J. L., Garteiser P. (2015). New imaging techniques for liver diseases. *Journal of Hepatology*.

[12] Agbim U., Asrani S. K. (2019). Non-invasive assessment of liver fibrosis and prognosis: an update on serum and elastography markers. *Expert Review of Gastroenterology & Hepatology*.

[13] van de Putte D. F., Blom R., van Soest H. (2011). Impact of Fibroscan® on management of chronic viral hepatitis in clinical practice. *Annals of Hepatology*.

[14] Chang P. E., Lui H. F., Chau Y. P. (2008). Prospective evaluation of transient elastography for the diagnosis of hepatic fibrosis in Asians: comparison with liver biopsy and aspartate transaminase platelet ratio index. *Alimentary Pharmacology & Therapeutics*.

[15] Castéra L., Vergniol J., Foucher J. (2005). Prospective comparison of transient elastography, Fibrotest, APRI, and liver biopsy for the assessment of fibrosis in chronic hepatitis C. *Gastroenterology*.

[16] Boursier J., de Ledinghen V., Zarski J. P. (2011). A new combination of blood test and Fibroscan for accurate non-invasive diagnosis of liver fibrosis stages in chronic hepatitis C. *The American Journal of Gastroenterology*.

[17] European Association for Study of Liver, Asociacion Latinoamericana para el Estudio del Higado (2015). EASL-ALEH Clinical Practice Guidelines: non-invasive tests for evaluation of liver disease severity and prognosis. *Journal of Hepatology*.

[18] Kattan M. W., Eastham J. A., Stapleton A. M. F., Wheeler T. M., Scardino P. T. (1998). A preoperative Nomogram for disease recurrence following radical prostatectomy for prostate cancer. *Journal of the National Cancer Institute*.

[19] Balachandran V. P., Gonen M., Smith J. J., DeMatteo R. P. (2015). Nomograms in oncology: more than meets the eye. *The Lancet Oncology*.

[20] Kim Y., Spolverato G., Ejaz A. (2015). A nomogram to predict overall survival and disease-free survival after curative resection of gastric adenocarcinoma. *Annals of Surgical Oncology*.

[21] Shim J. H., Jun M. J., Han S. (2015). Prognostic nomograms for prediction of recurrence and survival after curative liver resection for hepatocellular carcinoma. *Annals of Surgery*.

[22] Yoon W. S., Yang D. S., Lee J. A. (2017). Validation of nomograms for survival and metastases after hysterectomy and adjuvant therapy in uterine cervical cancer with risk factors. *BioMed Research International*.

[23] Notake T., Kobayashi A., Shinkawa H. (2017). Nomogram predicting long-term survival after the diagnosis of intrahepatic recurrence of hepatocellular carcinoma following an initial liver resection. *International Journal of Clinical Oncology*.

[24] Li Y., Ruan D. Y., Jia C. C. (2017). Surgical resection versus liver transplantation for hepatocellular carcinoma within the Hangzhou criteria: a preoperative nomogram-guided treatment strategy. *Hepatobiliary & Pancreatic Diseases International*.

[25] Wan G., Gao F., Chen J. (2017). Nomogram prediction of individual prognosis of patients with hepatocellular carcinoma. *BMC Cancer*.

[26] Xu X. L., Cheng H., Tang M. S. (2017). A novel nomogram based on LODDS to predict the prognosis of epithelial ovarian cancer. *Oncotarget*.

[27] Brockman J. A., Alanee S., Vickers A. J. (2015). Nomogram predicting prostate cancer-specific mortality for men with biochemical recurrence after radical prostatectomy. *European Urology*.

[28] Kawai K., Ishihara S., Yamaguchi H. (2015). Nomogram prediction of metachronous colorectal neoplasms in patients with colorectal cancer. *Annals of Surgery*.

[29] Kawai K., Ishihara S., Yamaguchi H. (2015). Nomograms for predicting the prognosis of stage IV colorectal cancer after curative resection: a multicenter retrospective study. *European Journal of Surgical Oncology*.

[30] Liu Y., Xue D., Zhang X. (2020). Nomogram for individualized prediction of hepatocellular carcinoma with portal vein tumor thrombosis on conservative treatment. *BioMed Research International*.

[31] Zhao E., Bai X. (2020). Nomogram based on microRNA signature contributes to improve survival prediction of clear cell renal cell carcinoma. *BioMed Research International*.

[32] Young K. A., Efiong E., Dove J. T. (2017). External validation of a survival nomogram for non-small cell lung cancer using the national cancer database. *Annals of Surgical Oncology*.

[33] Gao F., Zhang Q., Liu Y. (2019). Nomogram prediction of individual prognosis of patients with acute-on-chronic hepatitis B liver failure. *Digestive and Liver Disease*.

[34] Gao F., Li X., Wan G. (2018). Development and external validation of a prognostic nomogram for acute decompensation of chronic hepatitis B cirrhosis. *BMC Gastroenterology*.

[35] Luo M., Zheng H. Y., Zhang Y. (2015). A nomogram for predicting the likelihood of obstructive sleep apnea to reduce the unnecessary polysomnography examinations. *Chinese Medical Journal*.

[36] Pongchaiyakul C., Kotruchin P., Wanothayaroj E., Nguyen T. V. (2011). An innovative prognostic model for predicting diabetes risk in the Thai population. *Diabetes Research and Clinical Practice*.

[37] Semeraro F., Parrinello G., Cancarini A. (2011). Predicting the risk of diabetic retinopathy in type 2 diabetic patients. *Journal of Diabetes and its Complications*.

[38] Li W., Li Y., Zhang Z. (2016). Predictive nomogram of RAGE Genetic polymorphisms and metabolic risk factors for myocardial infarction risk in a han Chinese population. *Angiology*.

